# Body inversion effect in monkeys

**DOI:** 10.1371/journal.pone.0204353

**Published:** 2018-10-10

**Authors:** Toyomi Matsuno, Kazuo Fujita

**Affiliations:** 1 Department of Economics, Hosei University, Machida, Tokyo, Japan; 2 Department of Psychology, Graduate School of Letters, Kyoto University, Kyoto, Japan; National Institute of Child Health and Human Development, UNITED STATES

## Abstract

Humans visually process human body images depending on the configuration of the parts. However, little is known about whether this function is evolutionarily shared with nonhuman animals. In this study, we examined the body posture discrimination performance of capuchin monkeys, a highly social platyrrhine primate, in comparison to humans. We demonstrate that, like humans, monkeys exhibit a body inversion effect: body posture discrimination is impaired by inversion, which disrupts the configural relationships of body parts. The inversion effect in monkeys was observed when human body images were used, but not when the body parts were replaced with cubic and cylindrical figures, the positions of the parts were scrambled, or only part of a body was presented. Results in human participants showed similar patterns, though they also showed the inversion effect when the cubic/cylindrical body images were used. These results provide the first evidence for configural processing of body forms in monkeys and suggest that the visual attunement to social signals mediated by body postures is conserved through the evolution of primate vision.

## Introduction

Body posture is an important medium of communication for both humans and nonhuman primates (hereafter: NHPs). Nonverbal communication can replace or complement verbal or vocal communication and convey intent and emotional information in an explicit and implicit manner. Communicative gestures are widely observed in group-living primate species that build stable social relationships [1]. Therefore, precise perception of body postures is an ecologically important function of primate visual systems. Accordingly, recent studies on visual object perception in humans have revealed that body forms are processed in our visual system in a manner different from nonsocial visual objects [2–4], as are faces. For example, body forms more strongly capture visual attention [5, 6] and are perceived depending on spatial relationships among body parts [7, 8]; furthermore, specific brain areas that are activated are assumed to specialize in processing body forms [3, 9].

One prominent piece of evidence for specialized visual processing of body forms is the body inversion effect [10]. Unlike most other, nonsocial visual objects, inverted body postures tend to be much more difficult to discriminate or identify than upright ones. This body inversion effect suggests that our visual processing of bodies depends on the overall spatial relationships among body parts (configuration) rather than individual body parts [7, 11]. Previous studies have compared humans’ body inversion effect with the face inversion effect [10, 12], tried to clarify the critical requisite for the effect [13–16], and examined neural substrates [17–19].

In contrast to the extensive research on body perception in humans, we know little about how nonhuman animals perceptually represent bodies. Except for a few studies that examined recognition of bodies [20] and biological motion perception [21, 22], visual body processing has not been investigated behaviorally in NHPs. The comparative approach to body perception, however, is important to more fully understand both nonhuman and human visual processing of social stimuli.

Comparative studies on visual social perception in nonhuman animals have almost exclusively focused on face processing [reviewed in 23]. These studies have used face inversion and other paradigms to investigate whether the configural face processing mechanism is shared by humans and other primate species, but with inconsistent results. Whereas some studies reported similar face inversion effects [24, 25] or composite effects (i.e., horizontally aligned composite faces are more difficult to be recognized than misaligned ones) [26], in humans and NHPs, suggesting evolutionarily shared configural or holistic face processing mechanisms, others reported species differences [27, 28]. For instance, early studies by Rosenfeld and Van Hoesen [29] and Bruce [30] found that inversion did not induce disruption of face discrimination performance of macaque monkeys, and Parr, Winslow, and Hopkins [31] reported that inversion effects in monkeys were observed not only for facial pictures but also for nonsocial visual stimuli. Although it is difficult to unify and interpret these conflicting results, one possible explanation is that discrepancies may reflect the variety of procedures and stimulus configurations used in the experiments. Alternatively, species may truly differ in visual processing mechanisms for social stimuli. Some studies have directly compared the inversion effect in human and NHPs and among the latter using comparable testing methods and found species differences [27, 32]. For example, Weldon, Taubert, Smith, and Parr [33] used the Thatcher illusion, a phenomenon in which unusual local changes of facial features are harder to detect when the face is presented upside-down, and found that chimpanzees were sensitive to face orientation as humans were, but rhesus monkeys were not. The fact that studies with chimpanzees, the closest evolutionary relatives of humans, found results consistent with humans in contrast to mixed results for evolutionarily more distant primate species [23] may indicate phylogenetic variation in the configural processing of social stimuli. Therefore, studies using social stimuli other than faces would give an important new perspective on possible species homogeneity and heterogeneity of social perception mechanisms.

One intriguing reason for using bodies instead of faces in comparative studies on social perception is the species specificity of human bipedal locomotion. Facial morphological information about spatial relationships of facial parts (for example, a nose is below the eyes and above a mouth) is preserved across primate species including humans. This is consistent with the idea of an evolutionarily shared face processing mechanism. In contrast, a quadrupedal to bipedal shift in predominant body postures changes the spatial relationships of body parts (for example, from horizontal to vertical). Human bipedal postures maintain the vertical order of positions of body parts with the head at the top, followed by the arms, torso, and legs, whereas left-right positions of quadrupedal body parts vary depending on the postures and viewing angles. In addition, the perspective of humans, a terrestrial species, is relatively stable and we usually observe bodies of conspecifics in the same vertical plane, whereas arboreal species more commonly perceive conspecifics from various angles, including from above or below, which further increases the variation of spatial relationships among observed body parts. If the configural processing of body form in humans relied on such properties of bipedal postures, it might be evolutionarily discontinuous from other primates’ visual processing of bodies. Alternatively, however, the basic hierarchical structure of the body, with four limbs connected to a torso and a head between two upper limbs, is common to all primate species (although some species also have a tail). Given the widespread existence of a basic body configuration consisting of a trunk and locally movable limbs across many animal species, the adaptive value of configural body processing might be evolutionarily traceable back further than that of face processing.

Species differences in morphological constraints of the body also feature in the discussion on the mechanism of body perception. Studies on human configural body perception have debated the embodiment hypothesis. Several authors have reported that perception of another's body posture is influenced by the status of observer’s own body, suggesting a role for motoric body representations in configural body processing [e.g. 13, 34, 35]; however, recent research challenges this theory, finding independence of the observer's own bodily representation and processing observed bodies [36]. The question of whether visual body perception by monkeys, with fundamentally different locomotory representation, might share characteristics with that of bipedal humans has implications for the potential interaction between observers’ visual and own motoric body processing.

Comparison of visual body processing between human and nonhuman species is also important in considering the evolution of social cognition. Humans are highly social animals, leading to claims of their superiority over other primate species in terms of social cognitive skills. Several studies have reported less sensitivity in NHPs compared to humans to nonverbal communicative signals that are mediated by body postures [e.g. 37] and a limited capacity in NHPs for imitation and matching observed and own body forms [38–40]. Given the evidence for more sophisticated social cognitive abilities related to bodily social signals in humans compared to NHPs, the need for an accurate, elaborate body processing system might be more critical in the former. However, NHPs are not devoid of gestural communication abilities; indeed, some researchers have reported more flexible use of gestural signals than vocal ones [41]. Thus, skillful body perception might be crucial in the everyday social cognition of both humans and NHPs.

In this study, we examined the visual body perception of tufted capuchin monkeys (*Sapajus apella*) in comparison to humans. The capuchin monkey is a platyrrhine primate that separated from catarrhine species including humans about 40 million years ago [42]. They are arboreal quadrupeds and are known to have highly developed social cognitive abilities [43–47]. They also have been widely tested as a suitable nonhuman primate model of human visual perception [48–51]. Previous studies have shown that capuchin monkeys have face recognition abilities similar to humans, and they display the face inversion effect [52] as humans do. Therefore, capuchin monkeys were considered to be appropriate participants for the comparative examination of the body inversion effect.

**Fig 1:**
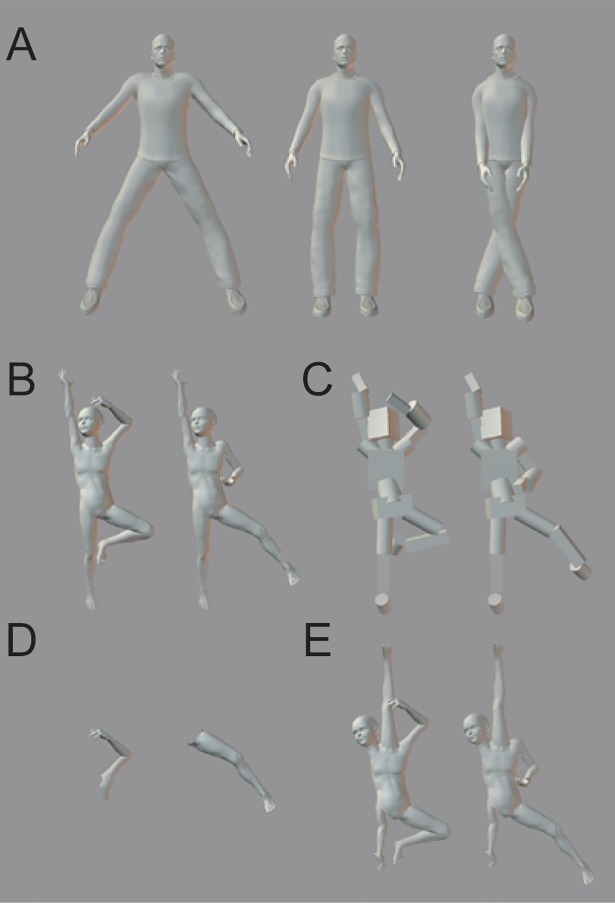
Examples of upright stimuli used in the experiments. (A and B) Whole body postures. (C) Cubic/cylindrical body postures. (D) Part body postures. (E) Scrambled body postures.

We conducted a series of comparative experiments examining the body inversion effect in monkeys and humans, to assess evolutionary continuity and possible specialization of the mechanism of human body perception. Monkeys and human participants were required to discriminate visual body images based on postural stimuli. We prepared upright and inverted versions of visual images of intact and transfigured human body forms created using 3D rendering software (Fig 1; see Method section). Participants performed a matching-to-sample task in which a figure was presented as a sample stimulus followed by two alternatives with different postures, one of which was identical to the sample. Matching accuracies for the trials in which the images were presented upright or upside-down (inverted) were compared to assess the body inversion effect.

In the pretest and Experiment 1, we examined a body inversion effect using intact human body stimuli. The second to fourth experiments were the control. Experiment 2 tested the effect of human-like appearance on the inversion effect. Each body part of the stimuli used in Experiment 1 was replaced by a cylinder or a box in Experiment 2. The stimuli preserve the gist configuration of the image but removed local feature information of the human body. Previous studies in humans have reported that body shape stimuli with such object-like appearance were not processed in the same way as human-like stimuli, suggesting the importance of human-like appearance in the visual processing of bodies [53, 54]. Experiment 2 examined whether human-like appearance would be the prerequisite for the body inversion effect or whether the visual analysis of gist configuration would be the only necessary condition for the process. In the former case, the object-like appearance may diminish the configural body processing, especially in monkeys, considering they had no visual experience nor a concept of humanoid robot bodies and lacked the knowledge-based processes that could guide body-specific visual processing. Experiments 3 and 4 investigated whether the inversion effects actually relied on the spatial relations (configuration of the body parts) or not. If the discrimination of upright isolated body parts (Experiment 3) or scrambled bodies (Experiment 4) was also advantageous to that of inverted ones, the observed inversion effect would be due to anisotropic local visual cues contained in some body parts rather than the canonical configuration of the body parts.

We hypothesize three cases to predict the results. First, configural body processing may be a trait of humans who are bipedal and have stable horizontal viewpoints, but not of monkeys. In this case, monkeys would discriminate bodies, like inanimate objects, depending on local visual features, resulting in the absence of inversion effects or invariant responses to the experiments regardless of whether the bodies are intact or not. Alternatively, humans and monkeys may share configural body processing, but it may work in a species-specific manner for each species. If configural body processing is constrained by species-specific locomotion and postures as the embodiment hypothesis implies, the body inversion effect with human body stimuli would be absent or much less significant in monkeys compared to humans, as hypothesized in the first case. The other assumption is that humans and monkeys may share more species-general configural body processing. In this case, monkeys may show body inversion effects with intact human body postures as humans do.

## General method

### Participants

Four tufted capuchin monkeys, Heiji (14-year-old male), Kiki (12-year-old female), Pigmon (10-year-old male), and Zilla (14-year-old female) participated in the experiments. All were captive-born and group-reared. They had previously performed various perceptual–cognitive tasks, including matching-to-sample tasks [55], but had no experience in discrimination tasks that used whole-body stimuli.

The monkeys lived in a group of seven at the Graduate School of Letters, Kyoto University, housed in 2 inter-connected indoor cages sized approximately 300 (width) × 180 (height) × 70 cm (length) and 160 × 180 × 70 cm. In the cages, steel decks, meshes and resin chains were placed and the monkeys were able to move around in the cages three-dimensionally. They were never deprived of food or water during the study. They earned a portion of their daily intake in the experiments and then received the remainder (primate pellets, fruits, vegetables, eggs) every afternoon. They had been trained to enter a transport box to move to the experimental room from the housing cages and return to the group immediately after the daily session.

Housing, care, and use of the monkeys adhered to the *Guide for the Care and Use of Laboratory Primates* of the Primate Research Institute, Kyoto University, Japan. The study was approved by the Animal Experiment Committee of the Graduate School of Letters, Kyoto University (No. 08–04).

Twenty-two undergraduate students (7 females and 15 males) ranging in age from 18 to 24 years (M = 19.5, SD = 1.4) also participated in the experiments; in return, they acquired course credits. They had no knowledge of both the face and body inversion effect prior to the experiments. All participants had normal or corrected-to-normal visual acuity.

The human experiments were conducted according to the Declaration of Helsinki and the experimental design was approved by the Research Ethics Committee of the Department of Economics, Hosei University (No. 2017-01-01). Written informed consent was obtained from each participant before conducting the experiment.

### Apparatus

The monkeys were tested in an operant box (approximately 45 × 45 × 45 cm) made of acrylic panels. One wall of each box had an opening (25 × 18 cm) behind which was a 15” TFT color monitor (Mitsubishi TSD-CT157-MN) equipped with a capacitive touch screen. This monitor presented stimuli generated from a Pentium-based computer and was also the input device for participants' responses. Two levers with an LED were attached below the opening. A universal feeder (Sanso S-100) delivered small pieces of food as a reward (apple or sweet potato) into a food tray on the left wall. White noise was used to mask external sounds.

Human participants were tested in an experimental room. They sat approximately 40 cm from the 23” TFT monitor (EIZO FORIS FS2333) with their heads steady on a chin and headrest. A USB game pad (Elecom JC-U2410T) was used to record their button press responses.

### Stimuli

The stimuli were 3D figures (Fig 1) created in gray scale using a 3D rendering software (Poser 6, Curious Labs Inc.) and outputted as 24-bit bitmap files. The images used in Experiments 3 and 4 (Fig 1D and 1E) were additionally edited using an image processing software (Photoshop CS, Adobe Inc.).

The base stimuli were bipedal human body forms. We used human body postures similar to those used in human body inversion studies [e.g. 10] to allow comparison of results of this study with previous ones. Note that the participant monkeys interact daily with human caregivers, and therefore it was assumed that they recognize human bodies. Monkeys show similar expertise in processing human faces as they do with conspecific faces, but not those of unfamiliar, heterospecific monkeys [52]. Furthermore, in some studies captive NHPs preferred and more accurately processed pictures of humans compared to conspecifics [56, 57], possibly because of their visual experiences with many humans and limited exposure to conspecifics. Therefore, the use of human body stimuli would be acceptable for the first examination of body inversion effect in monkeys.

### Procedure

A simultaneous matching-to-sample task was used for monkeys. Each trial started with the illumination of one LED in a lever (right lever for Heiji and Zilla, left lever for Kiki and Pigmon). When the monkey pressed the lever and held it down for 1 s, a sample stimulus appeared at the bottom center of the screen. After the monkey touched the sample stimulus, two alternatives appeared above the sample stimulus; one was identical to the sample, whereas the other was chosen from different postures in the same orientation (upright or inverted) as the sample. The vertical separation between the sample and the alternatives was 75 mm center to center distance, and the horizontal separation between alternatives was 126 mm, center to center. When the monkey touched the correct alternative (the same stimulus as the sample), a chime sounded for 0.5 s and a food reward (apple or sweet potato) was delivered to the feeder tray. An incorrect choice was followed by a 0.5-s buzzer sound and a 4-s time-out. The interval between presentation of the alternatives and touching one of them was recorded as the response time (RT).

Prior to the experiments, monkeys were trained and assessed in the matching-to-sample task with upright and inverted body stimuli (Fig 1A). Experiment 1 confirmed the inversion effect using body postures different from those used in training and pretest sessions (Fig 1B). Experiments 2, 3, and 4 used transfigured versions of the body stimuli images used in Experiment 1 (Fig 1C, 1D and 1E), and were run as control experiments to examine stimulus features underlying the observed inversion effect.

For comparison, human participants were tested on their discrimination of the body stimuli used with monkeys using a similar matching-to-sample procedure but with the following differences: sample stimuli were presented for only 500 ms to prevent a ceiling effect. After the disappearance of the sample stimulus, a 500 ms blank interval was inserted, and then two alternative stimuli appeared and remained on the screen until a response was made. Participants made a left-right selection using a left or right button on a gamepad at hand. No feedback was given after each trial. Prior to the experiments, participants were instructed to observe a briefly presented image and select the same image as the sample stimulus among two alternatives by pressing the left or right button as quickly and accurately as possible.

### Data analyses

The number of correct responses and mean RTs for correct trials were collected for each experimental condition in a session. For each condition within a session, RTs deviating more than 3 standard deviations from the mean were excluded as outliers following the modified recursive procedure by Selst and Jolicoeur [58]. Approximately 3.9, 2.8, 2.2, 3.7, and 2.8% of all trials with monkeys were excluded as outliers in the pretest and Experiments 1 to 4, respectively; similarly, 3.5, 4.6, 6.4, and 4.4% of all trials with humans were excluded as outliers in Experiments 1 to 4. The accuracy and RT data were analyzed using a generalized linear mixed model [59] with stimulus orientation condition and session as fixed factors and participant as a random factor. For the analyses of accuracy data, a binomial error distribution and logit link was assumed. The statistical significance of the inversion effect was tested by the Waldχ^*2*^ test. For the analyses of RT data, a Gaussian error distribution with identity link was assumed. The inversion effect of RT data was tested using the Wald *F* test with Kenward-Roger approximation of denominator degrees of freedom. Cohen's *f*^*2*^ was calculated based on marginal *R*^*2*^ statistics as the effect size [60–62]. All statistical analyses were conducted using R version 3.4 [63] and the lme4 and lmerTest packages [64, 65].

## Training and pretest

### Method

The body stimuli used in learning and pretest sessions were human male figures created in gray scale (Fig 1A). The height was 82 mm, and the width varied from about 18 mm to 52 mm depending on the posture. Three variations of arm positions and three variations of leg positions were prepared, resulting in a total of nine postures. The torso and head were invariant stimuli, such that monkeys could not use these parts as discriminative cues. Each posture was symmetrical and the arrangement of arms and/or legs differed from one another. Inverted stimuli were created by rotating the images by 180°.

A session consisted of 72 trials for both the training and test phases. The orientation of the stimulus, the postures of sample and foil (incorrect) stimuli, and the side of the correct comparison (left or right) in a trial was predetermined prior to the start of the session by pseudo-randomly selecting 72 trials without repetition from all the possible trial types (288 trial types: 72 sample-foil stimulus posture pairs × 2 stimulus orientations × 2 correct positions). The numbers of trials for upright and inverted conditions were not exactly equalized in a session due to an error in the experiment control program, but monkeys experienced an approximately equal number of trials in both conditions through the training and pretest sessions (49.7% inverted trials on average, S1 Fig).

The criterion for learning the matching-to-sample task in the training phase was set at above 80% correct matching in two consecutive sessions. The average number of sessions required to reach criterion in the training phase was 43 (70, 43, 23, and 36 sessions for Heiji, Kiki, Pigmon, and Zilla, respectively). Eight test sessions were conducted after criterion was reached, and performance in these was analyzed.

### Results

**Fig 2:**
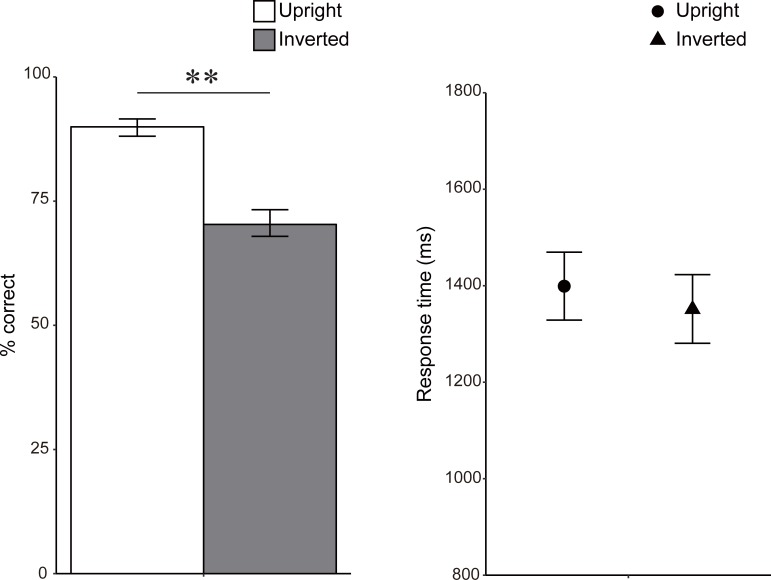
Mean percentages of correct responses and mean response times for correct trials in pretest sessions for monkeys. Error bars indicate bootstrapped 95% confidence intervals. **p < .01.

In the pretest results, the monkeys discriminated upright postures more accurately than inverted postures (Fig 2 and Table 1). The difference in the accuracy between the upright (89.8% correct on average) and inverted (70.6%) conditions in the pretest sessions was statistically significant, χ^*2*^(1) = 123.64, *p* = .00, *f*^*2*^ = 0.10, revealing that capuchin monkeys, like humans, are sensitive to the orientation of the image when they process body postures. RT data did not differ significantly between upright and inverted conditions, *F*(1, 58) = 0.85, *p >* .*10*, *f*^*2*^ = 0.00.

**Table 1:** Mean percentages of correct responses and mean response time data in eight pretest sessions for each stimulus orientation condition and each individual.

	% correct				Response time (ms)			
	Upright		Inverted		Upright		Inverted	
Monkeys	Mean	S.D.	Mean	S.D.	Mean	S.D.	Mean	S.D.
Heiji	87.4	8.6	70.7	9.1	1370.8	388.7	1415.3	366.1
Kiki	91.5	5.4	72.3	9.2	1398.5	248.8	1284.0	221.6
Pigmon	89.2	7.4	73.0	10.2	1474.8	407.0	1378.6	305.3
Zilla	91.7	7.4	65.2	14.0	1351.6	306.0	1330.6	306.3

## Experiment 1

### Method

To confirm whether the observed inversion effect in monkeys is a general phenomenon concerning body forms or dependent on specific postures or visual features learned in training, we conducted generalization tests using a new set of stimuli. In Experiment 1, we prepared figures of a naked young person standing on one leg with one arm extending upward, which were quite different from the images used in the pretest (Fig 1B). These postures are biomechanically possible for humans but are very uncommon for capuchin monkeys. The monkeys had never seen naked people, and caretakers probably never assumed these exact postures in front of the monkeys’ cages.

The height of the images was 71 mm and the width varied from 28 to 38 mm. Two variations of arm positions and two variations of leg positions were prepared (Fig 1B), yielding four postures. The postures were not symmetrical; therefore, mirror images of the upright and inverted stimuli were also prepared to cancel the left or right spatial deviation difference in discriminative cues between the upright and inverted conditions. The sample and foil stimuli differed in the position of either an arm, a leg, or both, and each sample and foil pair was tested four times in a session. Eight test sessions consisting of 96 trials each were presented immediately after the pretest. The left-right position of the correct comparison and the stimulus orientation condition (upright and inverted) was counterbalanced and pseudo-randomly intermixed.

### Results

**Fig 3:**
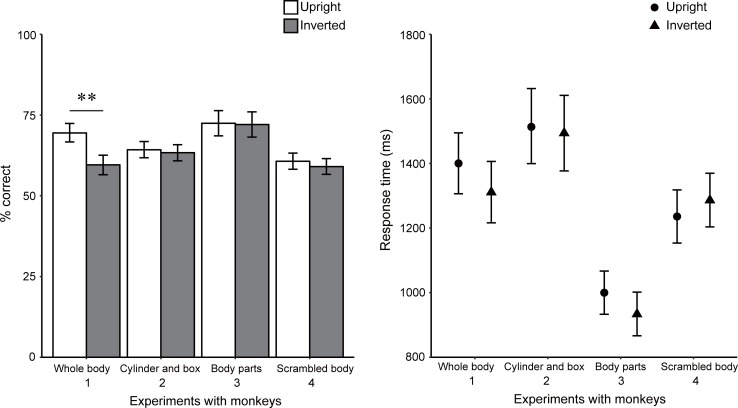
Mean percentages of correct responses and mean response times for correct trials in Experiments 1 to 4. Error bars indicate bootstrapped 95% confidence intervals. **p < .01.

The results replicated those in the pretest, showing body-inversion effects (Fig 3 and Table 2). The difference in accuracy between the upright (69.5% correct on average) and inverted (59.6%) conditions was statistically significant, χ^*2*^(1) = 32.74, *p* = .00, *f*^*2*^ = 0.02. RT data were not significantly different between upright and inverted conditions, *F*(1, 58) = 1.71, *p >* .*10*, *f*^*2*^ = 0.02.

**Table 2:** Mean percentages of correct responses and mean response time in eight test sessions for each stimulus orientation condition and each individual in Experiments 1 to 4.

		% correct				Response time (ms)			
		Upright		Inverted		Upright		Inverted	
		Mean	S.D.	Mean	S.D.	Mean	S.D.	Mean	S.D.
Experiment 1 (Whole body)									
	Heiji	64.3	12.7	59.4	11.4	1435.4	241.3	1407.3	312.5
	Kiki	65.9	15.7	58.9	14.3	1436.3	356.1	1331.8	305.2
	Pigmon	74.0	13.1	60.4	12.1	1405.3	327.4	1301.1	376.9
	Zilla	73.7	17.8	59.6	13.8	1324.0	478.0	1206.7	387.5
Experiment 2 (Cylinder and box)									
	Heiji	62.2	11.6	59.1	6.9	1558.4	314.8	1618.0	318.4
	Kiki	65.9	10.7	63.3	10.9	1411.9	278.7	1439.9	202.5
	Pigmon	63.8	16.9	65.6	13.9	1615.0	442.2	1574.8	240.7
	Zilla	65.1	15.1	65.4	12.1	1468.1	332.6	1347.2	208.4
Experiment 3 (Body parts)									
	Heiji	73.4	17.3	67.2	18.2	1015.2	200.4	871.0	283.8
	Kiki	69.5	15.8	73.4	17.6	961.7	286.5	917.2	210.2
	Pigmon	71.9	15.3	71.9	20.0	1035.3	141.2	1027.4	185.8
	Zilla	75.0	16.7	75.8	19.3	986.9	96.7	921.7	63.5
Experiment 4 (Scrambled body)									
	Heiji	57.3	12.3	59.9	6.8	1310.4	309.0	1264.2	220.7
	Kiki	61.2	13.2	59.4	10.1	1172.2	369.6	1303.3	369.8
	Pigmon	62.8	9.0	58.3	4.7	1181.1	346.9	1245.8	267.2
	Zilla	61.5	13.7	58.6	9.9	1279.1	400.2	1334.4	333.4

## Experiment 2, 3 and 4

### Method

Experiments 2, 3, and 4 were conducted as control experiments. In each experiment, the stimuli were prepared by transfiguring the stimuli used in Experiment 1.

Experiment 2 examined whether the human-like appearance of the stimuli affected visual body processing in monkeys. The postures of the figures were the same as those used in Experiment 1 except that each body part was replaced by a cylinder or a box, as shown in Fig 1C. This transfiguration did not change the gist configuration of the image but removed the local feature information of human body. The procedure was the same as that in Experiment 1.

Experiment 3 checked whether the individual body parts to be discriminated contained any orientation dependent cues or not (Fig 1D). The arm or leg that had been used as the discriminative cue was isolated from the other contextual body parts, which were invariant across the presentation of sample and foil stimuli in Experiment 1. The novel stimulus included either an arm or a leg, and the same parts of the different postures were selected, one as a sample and the other as a foil. Each sample and foil pair was tested four times as in the other experiments. Eight test sessions consisting of 32 trials each were presented.

Experiment 4 further tested the importance of spatial relationships among body parts in the monkeys' recognition of bodies by scrambling the positions of contextual body parts. Contextual limbs and head that did not feature as discriminative cues either in Experiment 1 or in this experiment were spatially scrambled with respect to the upright torso. A head was located at the position of an arm, the arm was at a leg’s position, and the leg was at the head’s position. Relative positions of the arm and leg that had been used as the discriminative cues to the torso were invariant and the same as the stimuli used in Experiment 2. The orientation of each stimulus (upright or inverted) was determined based on the limb positions that were not scrambled. Eight test sessions consisting of 96 trials each were presented.

### Results

Three control experiments using transfigured versions of the body images used in Experiment 1 confirmed that the observed advantage of upright posture discrimination was due to configural processing of apparent human body forms and was not an idiosyncratic effect resulting from biased responses to some spatial arrangement of local features (Fig 3 and Table 2). None of the following elicited an inversion effect: stimuli in which all body parts were replaced by cylinders and boxes (Experiment 2, Fig 1C, 64.3% and 63.4% correct responses for upright and inverted conditions, χ^*2*^(1) = 0.28, *p >* .*10*, *f*^*2*^ = 0.00), the parts without contextual limbs, the torso and the head (Experiment 3, Fig 1D, 72.5% and 72.1%,χ^*2*^(1) = 0.02, *p >* .*10*, *f*^*2*^ = 0.00), and scrambled body stimuli in which the contextual limbs and the head changed positions around the torso (Experiment 4, Fig 1E, 60.7% and 59.0%,χ^*2*^(1) = 0.85, *p >* .*10*, *f*^*2*^ = 0.00). RT data were not significantly different between upright and inverted conditions, *F*(1, 58) = 0.08, 1.85, and 0.75, *f*^*2*^ = 0.00, 0.03, and 0.01, *p*s > .10, for Experiments 2, 3, and 4, respectively, suggesting that the inconsistency between Experiment 1 and these experiments was not due to speed-accuracy trade-off.

## Experiments with humans

### Method

For comparative purposes, human participants also performed the visual discrimination tasks with the same stimuli used in experiments with monkeys and the presence or absence of the inversion effect was tested for each stimulus condition.

Participants completed four test sessions, each of which corresponded to a session in Experiments 1 to 4 with monkeys. The number of trials, stimuli, and the order of sessions were the same as in the experiments with monkeys. Before the test sessions, each participant was given a practice session of 10 trials, using the stimuli used in training sessions with the monkeys. No criterion for learning was set for the practice session, but none of the participants made more than one error.

### Results

**Fig 4:**
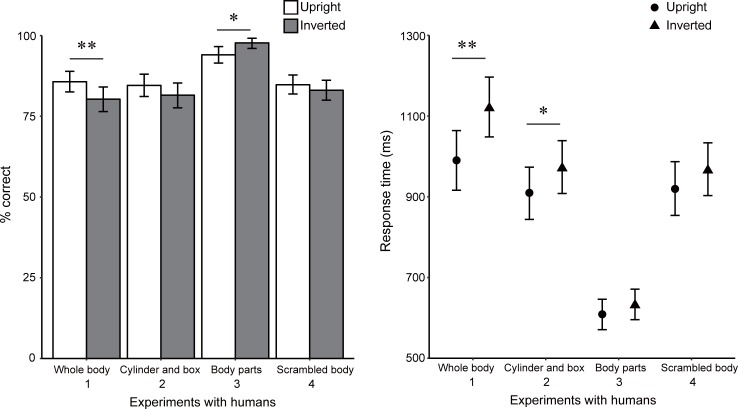
Mean percentages of correct responses and mean response times for correct trials in Experiments with humans. Error bars indicate bootstrapped 95% confidence intervals. *p < .05, **p < .01.

The experiment with humans using intact body stimuli replicated the results of previous body inversion studies with humans and those of monkeys in this study (Fig 4). The difference in accuracy between the upright (85.7% correct on an average) and inverted (80.3% correct on an average) conditions was statistically significant, χ^*2*^(1) = 11.26, *p* = .001, *f*^*2*^ = 0.01. The inversion effect was also significant for RTs on correct trials: participants responded faster for stimuli presented upright (990.4 ms on average) vs. inverted (1120.4 ms on average), *F*(1, 21) = 19.53, *p* < .001, *f*^*2*^ = 0.13.

Unlike monkeys, humans also tended to perform better (84.6% for upright condition and 81.5% for inverted condition) and respond faster (909.7 ms for upright condition and 971.5 ms for inverted condition) in the upright condition than the inverted condition when the body parts were replaced with cubes and cylinders. The difference in the accuracy was not statistically significant, χ^*2*^(1) = 3.58, *p* = .058, *f*^*2*^ = 0.00, but correct RTs were significantly different between conditions, *F*(1, 21) = 4.59, *p* = .044, *f*^*2*^ = 0.04.

Isolated parts were very accurately discriminated by humans. No upright advantage, but the opposite effect was observed (94.0% for upright condition and 97.7% for inverted condition, χ^*2*^(1) = 5.65, *p* = .017, *f*^*2*^ = 0.06). Correct RTs were not significantly different between conditions (608.8 ms for upright condition and 632.1 ms for inverted condition, *F*(1, 21) = 2.19, *p* > .10, *f*^*2*^ = 0.02). The isolated part discrimination was too easy for humans: almost 100% of responses were correct (24 data points among 44 participant-orientation condition pairs).

Discrimination performances for scrambled bodies were not significantly different between conditions (accuracy 84.8% for upright condition, 83.0% for inverted condition, χ^*2*^(1) = 1.16, *p* > .10, *f*^*2*^ = 0.00; correct RTs 919.4 ms for upright condition and 966.2 ms for inverted condition, *F*(1, 21) = 4.22, *p* = .053, *f*^*2*^ = 0.02), similar to the results of the monkeys.

## Discussion

In our experiments, monkeys exhibited the inversion effect when discriminating human body postures (pretest and Experiment 1). These results show that monkeys perceived the body images based on the spatial configuration of body parts rather than local features contained in individual body parts. Both deletion of contextual body parts and disruption of first-order spatial relationships by scrambling the positions of body parts abolished the effect (Experiments 3 and 4), further supporting our interpretation. The experimental order effect that potentially results in expertise in perceiving the discriminative cues contained in stimuli in later experiments could not explain the absence of inversion effects in control experiments, because the performance did not improve sequentially through the experiments. In fact, accuracy performance in Experiment 1 was not significantly different from that in Experiment 2, χ^*2*^ (1) = 0.45, *p* > .10, *f*^*2*^ = 0.00, and was better than that in Experiment 4, χ^*2*^ (1) = 14.14, *p* = .000, *f*^*2*^ = 0.00.

Comparative experiments with humans used the same stimuli and replicated the main results obtained with monkeys: humans also displayed the inversion effect with intact body stimuli and not with isolated parts or scrambled body figures. However, a species difference was found in the experiment with cubic/cylindrical stimuli. Monkeys showed no evidence of the inversion effect with the cubic/cylindrical stimuli, whereas humans performed better with upright than inverted cubic/cylindrical bodies, as in the experiment with intact human body postures. In addition, human participants showed significantly better performance in discriminating inverted body parts than upright body parts. Given the near perfect performance as described in the Results section, it may be a false positive result depending on the idiosyncratic ceiling effect. The other possible interpretation could be that the body part stimuli contained some orientation-dependent local cues that might be advantageous for the discrimination of inverted body parts. Minnenbusch, Suchan, and Daum [16] similarly demonstrated reversed inversion effects in humans' discrimination of headless body stimuli and indicated the possibility that a headless body could be perceived less configurally and the distinctive local features of the stimulus such as the position or shape of body parts may be more prominent for inverted stimuli. Such anisotropic local cues, if any, could work in a disadvantageous manner in discriminating upright bodies; therefore, this could not explain the observed inversion effect with intact body stimuli in our study.

The body inversion effect observed in this study is evidence that configural processing of body forms is shared by humans and platyrrhine monkeys. This suggests that configural body processing may have its evolutionary origin at least 40 million years ago, given the corresponding phylogenetic distance [42]. Alternatively, it may have evolutionarily converged considering the importance of social perception in both species. In either case, monkeys configurally perceive body postures similar to humans, and therefore, no obstructive bottleneck of sociocognitive capabilities appears to exist in the visual system of nonhuman primates, despite previous reports of species differences between humans and NHPs in reading gestural social signals [e.g., 37].

One notable aspect of our results is that monkeys exhibited a cross-species body inversion effect; in other words, quadrupedal monkeys perceived bipedal human postures in the same way as humans do. If observers' own motoric body representation played a critical role in configural body processing as the embodiment hypothesis implies, the inversion effect would be observed in a species-specific manner. Our results were not consistent with this view. Contrary to our results, previous studies with humans have reported body perception modulated by species and locomotive posture of the observed bodies. For example, Reed, Nyberg, and Grubb [35] examined body inversion effect in humans by manipulating the stimulus species (humans, dogs) and the typicality of postures (dog- or human-typical), and found the most marked effect with human figures with human-typical postures. In another study, Welsh, McDougall, and Paulson [66] used a stimulus-response compatibility paradigm and demonstrated that humans process pictures of animal bodies in bipedal postures in the same way as human pictures but not when the animal was quadrupedal. A critical difference between these studies and ours may be that our monkeys were exposed to human bodies every day, whereas most human participants in previous studies were probably less familiar with other species’ bodies. As mentioned earlier, studies have shown that captive NHPs process human faces similarly to those of conspecifics [52]. Our results imply that the same is true for body perception; configural body processing may be less dependent on constraints derived from the observer's own quadrupedal body representations, and it may depend on expertise or domain-specific visual tuning established through daily exposure in the same manner as configural face processing [67, 68]. This view is consistent with recent reports of intact body perception of individuals with atypical bodily representation due to deafferentation or limb dysplasia [13, 36].

The cross-species expertise for body processing may have adaptive value for animals in general. In contrast to face identification, which is crucial only for conspecific group members, body discrimination is important in both conspecific and heterospecific situations; therefore, more broadly tuned mechanisms not limited to own species' locomotion might be advantageous. For example, animals with notably different bodily constraints can engage in effective social interaction via bodily gestures; capuchin monkeys prefer to interact with human experimenters who imitate them [69], dogs can read human bodily gesture signals sometimes better than chimpanzees do [70] and dolphins imitate the actions of other familiar species, namely humans and seals [71]. Accurate body perception is also beneficial for nonsocial animals, as the prediction of future action from current bodily postures of prey or predators is advantageous for many species. From this perspective, future comparative studies with species with different bodily constraints and with different sociality would also be valuable for a better understanding of the evolution of body perception mechanisms.

In contrast to the commonly observed inversion effect with intact human body figures, the effect of cubic/cylindrical transfiguration was not consistent between monkey and human participants in our experiments. Humans displayed a smaller but significant inversion effect even when the body parts were replaced with cubic/cylindrical shapes without the human-like surface texture but which maintained the gist of an entire body form, whereas the monkeys’ performance was not sensitive to the stimulus orientation in this situation. Previous studies with humans have shown that biological (human-like) appearance is important for perceiving and recognizing moving agents [53, 54]. Appearance information could also affect visual processing of static body postures with similar modulatory mechanisms and play a role in promoting configural body processing. Considering this, the lack of an inversion effect in capuchin monkeys may be due to their ability to represent real objects from abstract expressions. We humans can easily conceive the three-dimensional human-like body figures from abstract and geometric two-dimensional images such as cubic/cylindrical stimuli, which might help to induce inversion effect with transfigured human figures. It may be harder for nonhuman animals to match such abstract and non-photographic images with real objects. For example, Parron, Deruelle, and Fagot [21] found no evidence that baboons are able to interpret point-light stimuli as human or monkey movements. They discussed that this failure may due to baboons' tendency to focus their attention on the local movement of dots and their limited ability to integrate degraded dot stimuli and encode them as iconic signs of natural three-dimensional objects. In another study, Tanaka [72] trained chimpanzees in a category learning task using photographs of natural objects, and reported that half of the participants failed to transfer their responses to generalization tests that used realistic sketches and line-drawings as stimuli. In our experiments, such an inability to represent the human body from degraded and abstract images would cause the absence of an inversion effect in Experiment 2.

In relation to this point, the presence of realistic facial information in naked body stimuli and its absence in the cubic/cylindrical body stimuli may be worth noting, considering the literature of human body inversion effects. Previous studies in humans have revealed that the head part particularly among the other body parts plays an important role in eliciting the body inversion effect [73, 74]. When the head part was removed, the body inversion effect disappeared [73], reversed [16], or diminished [75]. The cubic/cylindrical stimulus used in this study did not lack the head, but facial features such as eyes, a nose, and a mouth were not contained and the shape of the part was not a head-like ellipsoid. Such conditions may be adverse for head-dependent processes especially in monkeys who had no knowledge-based representation of a robotic head. In contrast, although the monkeys had probably never observed the naked human body and the stimuli used in Experiment 1 may have also been somewhat abstract for them, their general knowledge of the appearance of the human body learned from the observations of clothed people would be applicable to the naked body shapes with the help of preserved human-like head information.

In previous studies with humans, inversion effects in both face and body processing have been argued to support configural processing of these privileged social stimuli, distinct from inanimate objects, but whether configural processing of faces and bodies represent homogeneous or different processes has been debated. For example, it is conceivable that body processing shares with face processing initial mechanisms of lower order configural information such as spatial relations among parts, but lacks some later stages of integration (holistic processing) which is supposedly present in face perception [7, 76]. However, recent studies have accumulated evidence of holistic processing in body perception similar to face perception [77, 78], which means that a body is perceived not only as the spatial relationship among body parts but also as an undecomposed whole. In this context, our results on the body inversion effect provide evidence that humans and capuchin monkeys share configural processing of at least basic relational information. For investigations on more integrative stages of configural processing of body information, further comparative studies of nonhuman animals in different experimental paradigms used to test holistic configural processing in humans, such as testing the composite effect of body postures, are needed.

Our work is limited in several ways, which future work can address. First, in our study, the number of both the monkey participants and the tested species was limited. Our conclusion was based on the behavioral data of four monkeys who had lived both with group mates and human caretakers in captive environment. Therefore, it is a worthy future task to determine how visual processes are shared between monkeys with a variety of rearing histories and visual experiences, including wild capuchins. Furthermore, both humans and capuchin monkeys are known to be highly social primate species. To elucidate the evolutionary origin of body perception, comparative studies in other primate and nonprimate species of varying sociality and morphological body constraints are needed.

In addition, we examined monkeys' body perception focusing only on posture differences of the same individuals, but recent human studies have revealed that bodies contain much richer social information, such as identity of the owner, individual characteristics (including sex, age, power, and attractiveness), emotional expressions, and interactive intentions [79, 80]. Therefore, future comparative studies could usefully examine whether NHPs extract social information from bodies in the same manner as humans, and how configural and featural body processing mediate recognition.

## Conclusion

In this study we aimed to shed light on the characteristics of body perception of NHPs in comparison with humans. We examined whether monkeys demonstrate the body inversion effect, which has been argued to be the behavioral hallmark of configural body processing. Like humans, monkeys discriminated upright bipedal human body postures more accurately than their inversions. The observed effects were not due to any orientation-dependent local cues contained in individual body parts. There were also slight species differences: monkeys did not exhibit any inversion effect when human-like appearance was removed from the stimuli, whereas human performance was still orientation-dependent in such a situation. In sum, these results suggest that despite their different quadrupedal and bipedal locomotive bodily constraints, monkeys and humans share the percept of body postures according to their basic configural properties.

## Supporting information

S1 Fig Percentages of correct responses in the training and pretest sessions for four monkeys. Each symbol represents overall performance (square) or performance in the upright (circle) or inverted (triangle) condition in a session. The dashed lines indicate the percentage of trials in the inverted condition.
(PDF)

S1 File Data set. (CSV)
