# Supplementary material for: Body inversion effect in monkeys
Source: PLoS One. 2018 Oct 10;13(10):e0204353. doi: 10.1371/journal.pone.0204353 (PMC6179237; doi:10.1371/journal.pone.0204353)
Supplement: S1 Fig — Each symbol represents overall performance (square) or performance in the upright (circle) or inverted (triangle) condition in a session. The dashed lines indicate the percentage of trials in the inverted condition. (PDF) [file pone.0204353.s001.pdf]

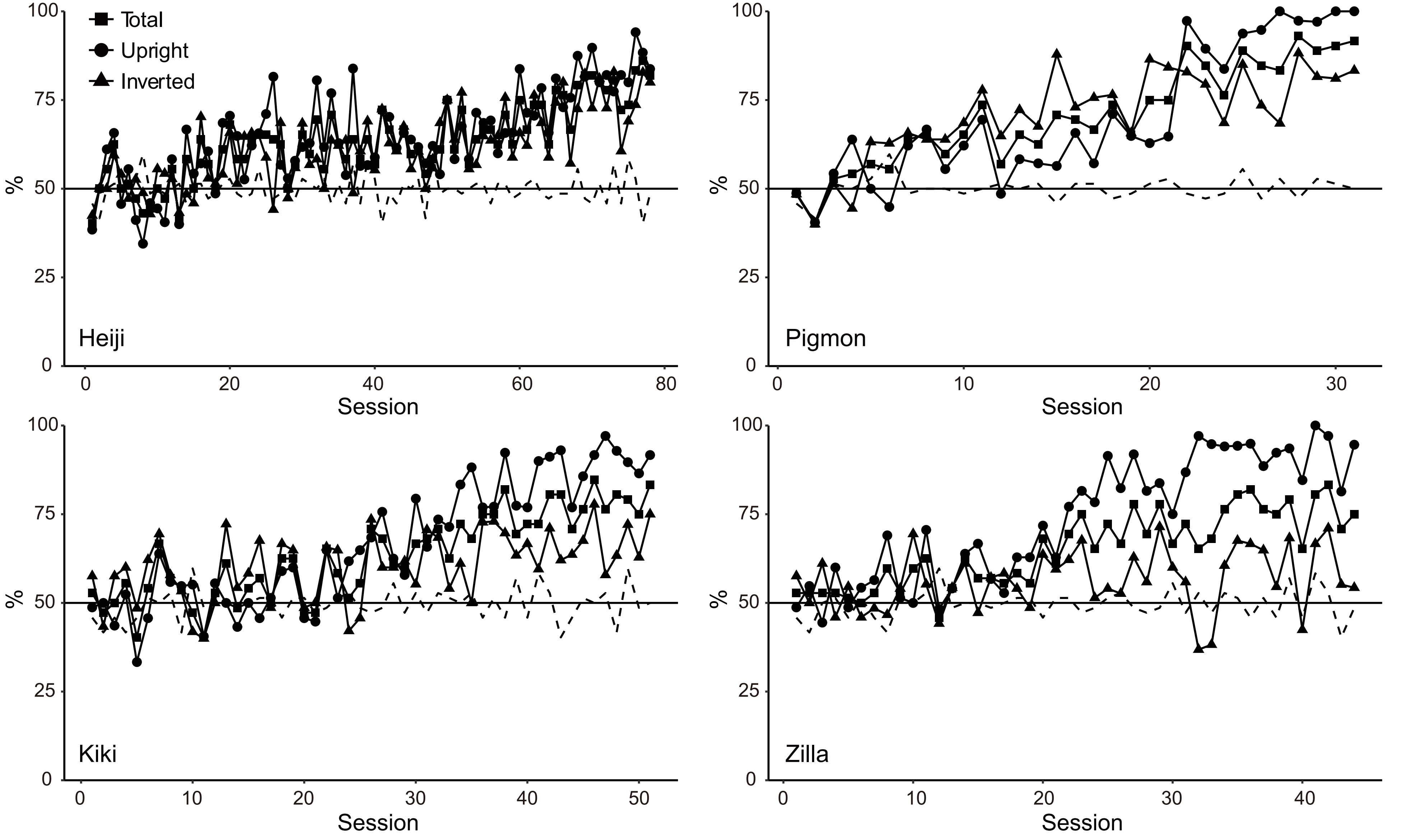

Supplementary Figure 1. Percentages of correct responses in the training and pretest sessions for four monkeys. Each symbol represents overall performance (square) or performance in the upright (circle) or inverted (triangle) condition in a session. The dashed lines indicate the percentage of trials in the inverted condition.
